# Thin Copper Plate Defect Detection Based on Lamb Wave Generated by Pulsed Laser in Combination with Laser Heterodyne Interference Technique

**DOI:** 10.3390/s24103103

**Published:** 2024-05-14

**Authors:** Xinhao Wang, Zhaojiong Zhu, Guqing Guo, Xiaocong Sun, Ting Gong, Yali Tian, Yueting Zhou, Xuanbing Qiu, Xiaohu He, Huiqin Chen, Christa Fittschen, Chuanliang Li

**Affiliations:** 1 School of Materials Science and Engineering, Taiyuan University of Science and Technology, Taiyuan 030024, China; b20201491011@stu.tyust.edu.cn (X.W.); chenhuiqin@tyust.edu.cn (H.C.); 2 Shanxi Province Engineering Research Center of Precision Measurement and Online Detection Equipment, Shanxi Center of Technology Innovation for Light Manipulations and Applications, School of Applied Science, Taiyuan University of Science and Technology, Taiyuan 030024, China; zhuzhaojiong@stu.tyust.edu.cn (Z.Z.); guqingguo@tyust.edu.cn (G.G.); sunxiaocong@tyust.edu.cn (X.S.); gongting@tyust.edu.cn (T.G.); tianyali@tyust.edu.cn (Y.T.); zyt@tyust.edu.cn (Y.Z.); qiuxb@tyust.edu.cn (X.Q.); xiaohuhe@tyust.edu.cn (X.H.); 3 CNRS, UMR 8522-PC2A—Physicochimie des Processus de Combustion et de l’Atmosphère, Université Lille, F-59000 Lille, France; christa.fittschen@univ-lille.fr

**Keywords:** laser ultrasonic, laser heterodyne interferometer, Lamb wave, thin copper plate, continuous wavelet transform

## Abstract

Thin copper plate is widely used in architecture, transportation, heavy equipment, and integrated circuit substrates due to its unique properties. However, it is challenging to identify surface defects in copper strips arising from various manufacturing stages without direct contact. A laser ultrasonic inspection system was developed based on the Lamb wave (LW) produced by a laser pulse. An all-fiber laser heterodyne interferometer is applied for measuring the ultrasonic signal in combination with an automatic scanning system, which makes the system flexible and compact. A 3-D model simulation of an H62 brass specimen was carried out to determine the LW spatial-temporal wavefield by using the COMSOL Multiphysics software. The characteristics of the ultrasonic wavefield were extracted through continuous wavelet transform analysis. This demonstrates that the A_0_ mode could be used in defect detection due to its slow speed and vibrational direction. Furthermore, an ultrasonic wave at the center frequency of 370 kHz with maximum energy is suitable for defect detection. In the experiment, the size and location of the defect are determined by the time difference of the transmitted wave and reflected wave, respectively. The relative error of the defect position is 0.14% by averaging six different receiving spots. The width of the defect is linear to the time difference of the transmitted wave. The goodness of fit can reach 0.989, and it is in good agreement with the simulated one. The experimental error is less than 0.395 mm for a 5 mm width of defect. Therefore, this validates that the technique can be potentially utilized in the remote defect detection of thin copper plates.

## 1. Introduction

Currently, thin copper plates, renowned for their remarkable thermal and electrical conductivity, excellent ductility, and inherent resistance to corrosion, are indispensable in various fields such as construction, automotive manufacturing, electrical components, and marine applications, showcasing their crucial role in modern technology and industry [1,2,3,4]. However, the copper plate may exhibit slight surface imperfections generated during the manufacturing process [5]. Timely detection of these tiny or micro defects could prevent their progression into larger structural and mechanical integrity issues. Therefore, it is significant to develop real-time quantitative measurement and assessment of micro defects on the surface of copper strips [6].

Several non-destructive testing techniques have been established, including penetrant testing, eddy current testing, ultrasonic testing, radiographic testing, and magnetic particle inspection. Nonetheless, there are certain limitations for these methods [7,8,9,10,11]. For instance, penetrant testing is affected by surface roughness and is not suitable for porous materials. Eddy current testing is sensitive to magnetic permeability changes, making it effective only for conductive materials and not effective for defects parallel to the surface. Ultrasonic testing principally relies on contact transducers and is improper for in-situ harsh environments. Radiographic testing is expensive and inefficient for cracks with special orientation. Magnetic particle inspection fails to detect low-permeability materials or deeply situated defects in casting and requires a sample with a smooth surface. Compared to these methods, laser ultrasonic testing possesses a lot of advantages, including non-contact operation, a high-frequency bandwidth, the ability to generate multi-mode ultrasound, independence from the electronic or magnetic properties of the testing materials, and a flexible laser shape for excitation [12,13,14,15]. Consequently, laser ultrasonic testing is increasingly utilized for determining the properties and identifying defects of materials.

A Lamb wave (LW) is a guided wave that travels through thin plates, with characteristics such as low attenuation and the capability to cover substantial distances [16,17,18]. The generation of LWs can be categorized into two primary techniques: direct-contact methods [19,20,21,22,23] and non-contact ones [24,25,26,27,28]. There are various approaches for receiving LW signals. Yu et al. utilized piezoelectric wafer active sensors to both generate and receive LWs propagating within a plate-like structure [29]. Paul Wilcox et al. employed electromagnetic acoustic transducers to produce and collect LWs in steel and aluminum plates with thicknesses from 5 to 10 mm [30]. Air-coupled sensors are also utilized to excite and receive LWs in composite plates to achieve the baseline-free delamination inspection technique of composite plates [31]. The fiber Bragg grating sensor is renowned for its high sensitivity in detecting LWs [32]. However, these techniques require the detector and sample to be in close proximity or in direct contact for receiving the LW. This would restrict their applicability in remote or harsh environments. To address this constraint, Naoki Hosoya et al. employed a scanning laser Doppler vibrometer to detect LWs induced via laser on an aluminum plate [33]. Additionally, a two-wave mixing interferometer was used to detect ultrasound and characterize the grain-size defect distribution of aluminum [34].

The primary methods for processing LW signals involve time-domain, frequency-domain, and time–frequency domain analysis. The time-domain method is characterized by its simplicity and intuitiveness, but it is susceptible to environmental interference and demands a strict experimental condition. Frequency-domain analysis is a straightforward and effective method, particularly for dealing with extensively damaged specimens. However, a LW as a transient signal exhibits dispersion characteristics and displays non-stationary features. Consequently, it is crucial to understand the frequency-domain characteristics of the LW signal at different times for defect detection. Furthermore, time–frequency domain analysis has been proposed to retrieve more accurate and comprehensive information. The time–frequency domain method frequently utilizes techniques such as short-time Fourier transform (STFT), wavelet transform, Hilbert–Huang transform (HHT), Wigner–Ville Distribution (WVD) and Cohen Class Distributions [35,36,37,38]. STFT exhibits limited time resolution due to its fixed window width. A broader window enhances frequency resolution but diminishes time resolution, while a narrower window improves time resolution at the expense of frequency resolution [39]. Although the HHT shows promise as a valuable tool for extracting features from non-stationary signals, it encounters challenges in system identification due to the generation of spurious modes and susceptibility to mode mixing [40]. Conversely, the computational complexity of the WVD renders it impractical for large datasets due to its complexity [38]. Cohen Class Distributions may introduce cross-term interference, particularly problematic when analyzing multi-component signals [38]. Comparatively, the continuous wavelet transform (CWT) offers excellent resolution in both the time and frequency domains. Utilizing the complex Morlet wavelet provides the precise measurement of frequency localization for signals with both fast and slow oscillations. This adaptability is achieved through a flexible window that narrows for high frequencies and widens for a low-frequency [41].

This work presents a laser ultrasonic inspection system based on an all-fiber heterodyne interferometer. A 3-D model of an H62 brass specimen was simulated by COMSOL Multiphysics software (version 6.0). In the simulation, a LW was generated by a pulsed laser, and the transmitted and reflected wave signals were investigated by the spatial-temporal wavefield. The CWT was employed to seek the optimum frequency of the LW with maximum energy. In the experiment, the displacement of receiving spots was observed with a time resolution of 1 ns. A comparison test was carried out for both a defect-free sample and a defect sample. The frequency component at 370 kHz was extracted to discern the defects. Both the location and width of defects were determined through the time difference of the reflected and transmitted signals, respectively.

## 2. Finite Element Method

### 2.1. Material Parameters

A three-dimensional model for ultrasonic wave inspection was established using the simulation software COMSOL Multiphysics. The simulation of the ultrasonic wave was generated by a pulsed laser on a specimen corresponding to the LW. The simulating sample was copper, characterized as a linear elastic material with the same size as the testing specimen.

**Figure 1:**
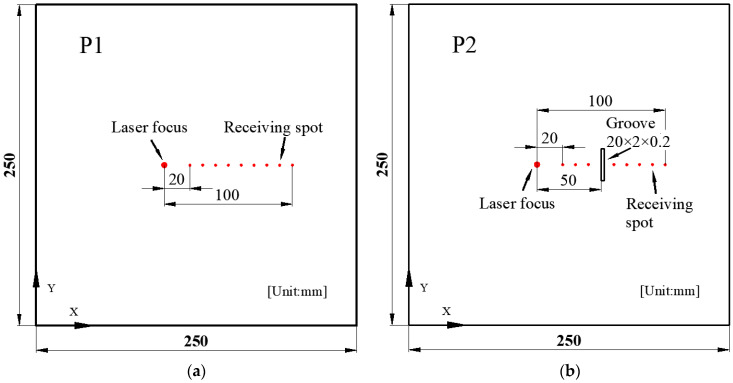
The tested copper plate. (**a**) Defect-free sample P1. (**b**) Defect sample P2.

The copper plate specimen has dimensions of 250 mm × 250 mm × 0.5 mm. To simulate surface defects on the copper plate, a groove is machined on the surface, as depicted in Figure 1. For comparisons, a defect-free copper plate is labeled as P1, and a copper plate with a defect is designated as P2 with grooves of 20 mm × 2 mm × 0.2 mm. The laser focal point is fixed in the experiment, while the receiving spot moves in parallel with a step size of 2 mm. The distance from the laser focus to the receiving spot increases from 20 mm to 100 mm. The material properties are listed in Table 1.

Low reflection boundary conditions are implemented to mitigate the boundary reflection and ensure accurate wave propagation modeling. This approach minimizes wave reflection at boundaries, allowing for the isolation and analysis of the primary surface. The pulsed laser excitation is treated as a boundary condition loaded on the surface of the material. This boundary condition includes both the thermal effect of the laser and the temporal and spatial distribution functions of the laser pulse. The thermal effect is simulated via heat equation [42,43,44].

### 2.2. Displacement Field Analysis

**Figure 2:**
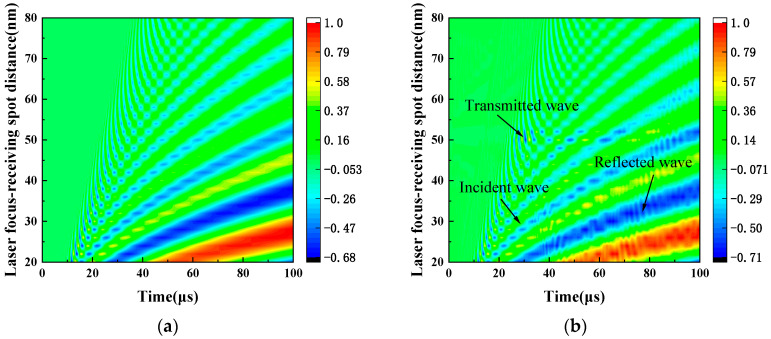
Laser LW spatial-temporal wavefield. (**a**) Defect-free copper plate P1. (**b**) Defect copper plate P2.

As shown in Figure 2, the number of oscillations in the wavefield signal over time rises with increasing laser focus-receiving spot distance. This observation indicates the separation of frequency components and the emergence of distinct propagation patterns. The broad frequency spectrum of the laser-generated LW leads to distinct propagation patterns dependent on the excitation-reception distance. At close proximity, the limited propagation distance results in overlapping frequency components, forming a composite vibration pattern. Nonetheless, the various frequency components begin to separate gradually with the distance increase between excitation and reception points, revealing their unique propagation characteristics.

LW signals exhibit a distinct frequency-dependent propagation pattern. At a given location, high frequency components arrive first, followed by the lower-frequency components carrying more energy. This sequential arrival stems from the inherent composition of laser-generated LW signals, which are primarily low-frequency. Additionally, the perpendicular orientation of the receiving beam to the plate enhances its sensitivity to out-of-plane LW displacements, further increasing the prominence of low frequency A_0_ mode components in the received signal [45]. The observed wavefield signal characterizes low energy and fast wave speed for high-frequency components, and high energy and slow wave speed for low-frequency components. It aligns with the theoretical prediction A_0_ mode varying with frequencies. With comparisons of Figure 2a,b, spatial-temporal wave field signal analysis reveals the distinct reflection and transmission of the incident LW at the defect site. CWT is implemented to analyze the laser-generated LW signals for further extraction of the defect characteristics.

**Figure 3:**
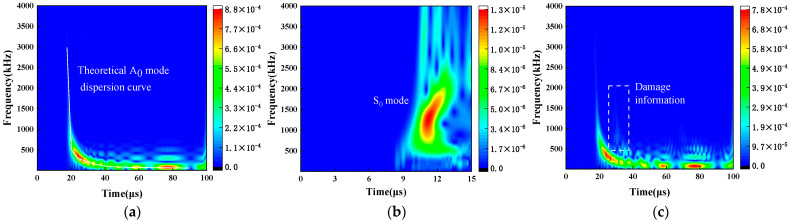
Time–frequency graph after CWT at a laser focus-receiving spot distance of 40 mm. (**a**) Copper plate P1. (**b**) Copper plate P1 at 0–15 μs. (**c**) Copper plate P2.

As shown in Figure 3, a time–frequency analysis is conducted through CWT. The Morlet wavelet is chosen as the mother wavelet. The LW spectrum primarily distributes in the range of 0 to 2 MHz. It is important to note that higher-order modes have not been detected within this frequency range. In Figure 3a, the time–frequency representation demonstrates a strong resemblance to the theoretical curve of the A_0_ mode. An energy peak is evident, occurring at 24.5 μs. In Figure 3b, the time–frequency representation is generated by the LW displacement from 0 to 15 μs based on CWT. A distinct signal associated with the S_0_ mode displays around 1.5 MHz, which can be found in Figure 3c. The energy with respect to the defect is located at approximately 370 kHz, which corresponds to maximum intensity [28]. It is noteworthy that the time–frequency analysis lacks the precise spatial resolution for defect localization [46].

### 2.3. Lamb Wave Dispersion Characteristics

**Figure 4:**
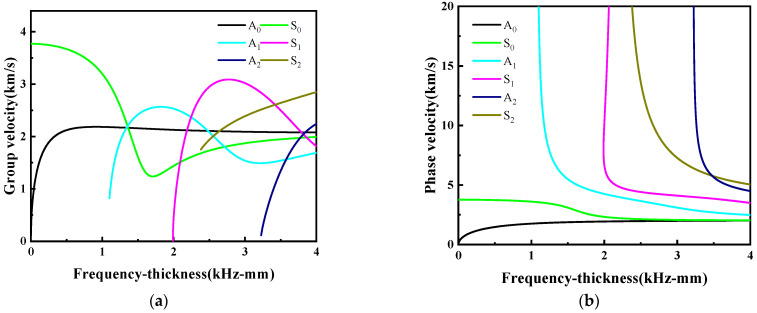
Dispersion curves of the H62 brass. (**a**) Group velocity dispersion curves. (**b**) Phase velocity dispersion curves.

Dispersion curves of H62 brass are shown in Figure 4. The dispersion curves show that at frequency thickness lower than 1 MHz-mm, only the fundamental A_0_ and S_0_ Lamb modes exist. When frequency thickness goes higher, more LW modes (such as A_1_, A_2_, S_1_ and S_2_) appear. The velocities of most modes have a high dependence on frequency. Therefore, in the non-destructive testing of Lamb, small frequency thickness should be used for detection.

## 3. Experiment Setup

**Figure 5:**
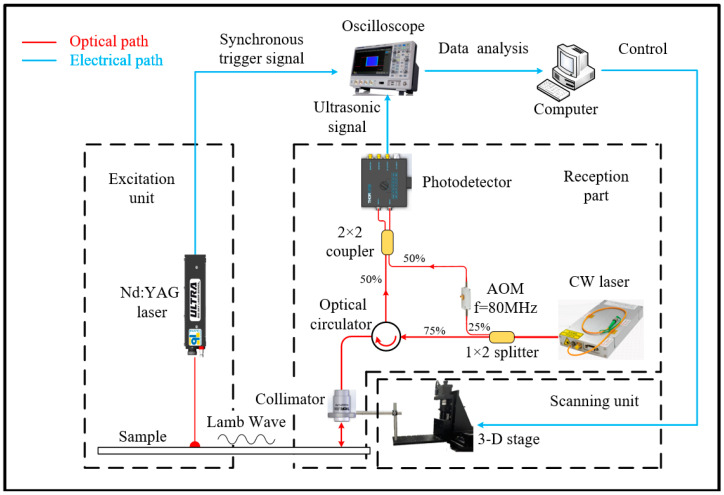
Experimental setup of laser ultrasonic generation and detection system.

As illustrated in Figure 5, the experimental setup consists of the laser ultrasonic generation and detection system, which includes an excited pulse laser, an all-optical fiber heterodyne interferometer for LW detection, and a scanning subsystem. The Nd: YAG laser (Quantel, Ultra, 1064 nm, 6 ns, Les Ulis Cedex, France) irradiates the surface of the H62 brass sample. Simultaneously, the laser controller generates a signal to trigger the oscilloscope and synchronize the laser heterodyne interferometer. For the laser heterodyne interferometer, a continuous wave fiber laser (Precilasers, FL-SF-1550-S, Shanghai, China) is divided into two beams via a fiber beam splitter (1:3). One beam serves as the reference light, passing through the acousto-optic modulator at 80 MHz (Qingjin, G-1550-80-L-B-T-AA-A-Y-L, Shanghai, China). The remaining beam (75%) is directed onto the surface of the copper plate, and part of it is reflected back by the sample surface. The reflected beam carrying the ultrasonic signal is collected by a reflective collimator (Thorlabs, RC02APC-P01, NJ, USA) [47,48]. Then, it is transmitted to the optical circulator and combined with the reference laser by a 2 × 1 (50/50) fiber coupler onto a balanced amplified photodetector (Thorlabs, PDB450C-AC). When the two signals are combined, their frequencies mix, and the output beat signals of the photodetector are digitally displayed and stored by the oscilloscope (SIGLENT SDS2504X Plus, Shenzhen, Guangzhou, China). For precise and automated scanning, the collimator is mounted on a 3D-stage equipped with stepper motors. Its position can be adjusted by using a computer.

During the experiment, the laser’s single pulse energy is set to 50 mJ. This setting ensure effective excitation of ultrasonic signals through the thermoelastic mechanism and guarantees the integrity of the copper plate [49]. Thirty-two pulses of repetition were averaged at the same location to improve the signal-to-noise ratio. These ultrasonic signals acquired via digital oscilloscope were processed by custom-written MATLAB software (version 2021a). IQ demodulation is used to extract the ultrasonic signal and generate time-resolved surface displacement resulting from the propagation of LWs [50,51]. Subsequently, the displacement was analyzed in both the time and frequency domains. The time resolution can achieve 1 ns. In the experimental samples, five samples with different defect widths were selected, with widths of 2 mm, 4 mm, 5 mm, 6 mm, and 8 mm, respectively. Their positional dimensions are the same as those shown in Figure 2b. The distance between the laser focus and the left side of the defect is 50 mm.

## 4. Results and Discussion

**Figure 6:**
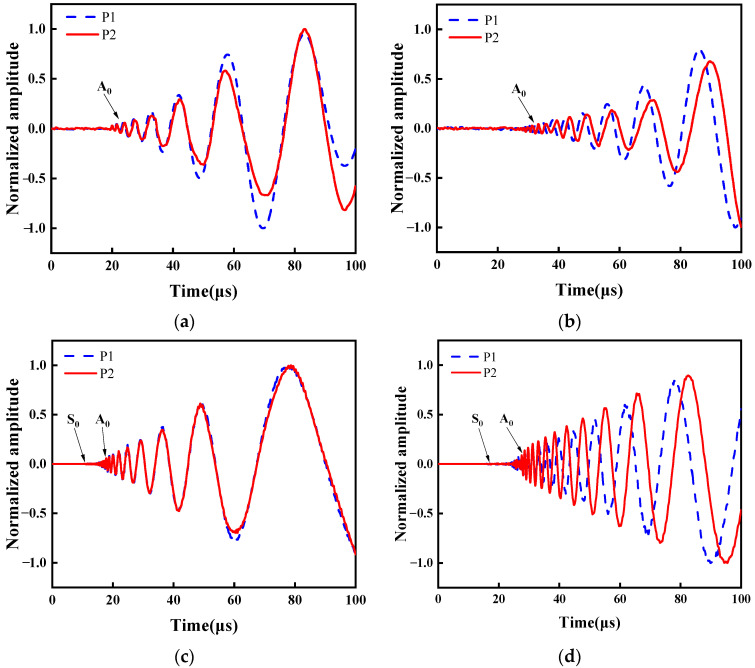
LW time-domain signal. (**a**) Experiment results at a laser focus-receiving spot distance of 40 mm. (**b**) Experiment results at a laser focus-receiving spot distance of 60 mm. (**c**) FEM simulations at a laser focus-receiving spot distance of 40 mm. (**d**) FEM simulations at a laser focus-receiving spot distance of 60 mm.

LW time-domain signals are presented in Figure 6. This demonstrates the evident displacement with respect to the dispersion characteristic [52]. As plotted in Figure 6a, the displacement amplitude is low between 0 μs and 20 μs. From 20 μs to 100 μs, displacement amplitude increases gradually. Around 10 μs, the S_0_ mode was not observed. The S_0_ mode is primarily in in-plane displacement, with minor displacement perpendicular to the surface. Therefore, it could be measured by laser interferometer. The A_0_ mode is evident from the prominent dispersion observed in Figure 6a. The displacement at a laser focus-receiving spot distance of 40 mm for plates P1 and P2 is synchronized in Figure 6a, whereby a noticeable time delay occurs at a laser focus-receiving spot distance of 60 mm in Figure 6b. This reveals no significant displacement variation on the P2 plate attributed to the reflection of the defect signal, so it is difficult to recognize defects by LW displacement. Nevertheless, this does not imply that the reflected signal has not been generated due to a defect. Further analysis of the LW signal in the time domain is imperative for extracting the defect signal. Figure 6c,d depicts the LW time-domain signals simulated using FEM. S_0_ mode and A_0_ mode can be found. These FEM results are consistent with the corresponding experimental results, thus confirming the accuracy of the experimental findings.

**Figure 7:**
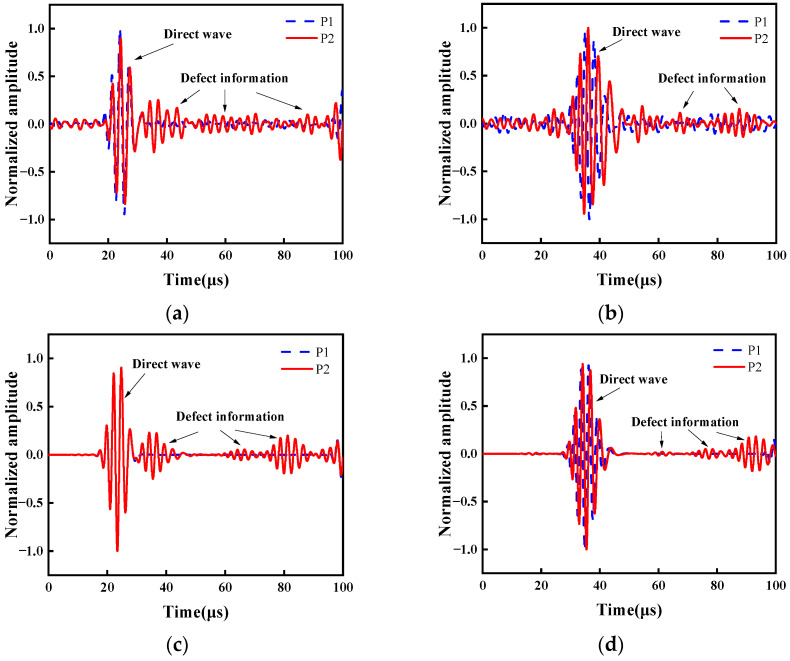
LW signal of 370 kHz extracted by CWT. (**a**) Experiment results at a laser focus-receiving spot distance of 40 mm. (**b**) Experiment results at a laser focus-receiving spot distance of 60 mm. (**c**) FEM simulations at a laser focus-receiving spot distance of 40 mm. (**d**) FEM simulations at a laser focus-receiving spot distance of 60 mm.

To effectively derive the defect characterization, a frequency of 370 kHz is extracted from the wavelet coefficient spectra of the signals for both the defect-free and defect sample. LW signals are analyzed using CWT, as shown in Figure 7. The direct waves are the same in both P1 and P2 before passing the defect. Therefore, this enables the identification of the location of defect via the presence of the reflected wave. The Hilbert transform is utilized to extract the envelope of the LW. When the envelope amplitude reaches its maximum value, it indicates the time of arrival of Lamb waves at the receiving spot. According to theory, the group velocity of the A_0_ mode at 370 kHz is 1747.1 m/s in the copper plate. In the experiment, the average velocity of the direct wave is 1756.7 m/s, while the average velocity of the reflected wave is 1759.6 m/s. The velocity error is only 0.94%. Based on the experimental results, the specific location of the defect is identified at 50.08 mm. The propagation distance and defect location are determined through a single frequency. In Figure 7b, the direct wave A_0_ mode discrepancy is observed in both P1 and P2. This is because of the interaction of the LW and defect, and it induces changes in both their paths and modes. Consequently, the waveforms no longer align or overlap. Therefore, the transmitted signals, compared to the reflection signals of the LW, demand more complex processing. Figure 7c,d depicts the LW signal of 370 kHz extracted by CWT simulated using FEM. These FEM results are consistent with the corresponding experimental results, and they are not affected by noise interference.

**Figure 8:**
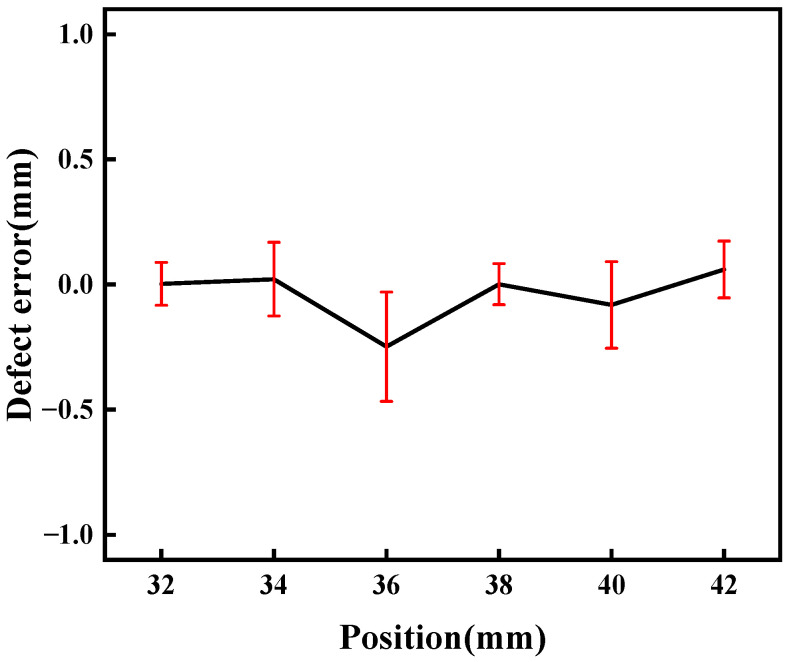
Defect errors at various laser focus-receiving spot distances.

The relative error of the defect measured at various laser focus-receiving spot distances is plotted in Figure 8. It varies for determining the defect wave when the receiving point is close to the defect. Therefore, six measuring points were selected to improve the accuracy, and the average error is reduced to 0.14%. The minimum error is derived at a distance of 12 mm from the defect, and the largest one is obtained at a distance of 14 mm. This indicates the feasibility of determining the defect location using the reflection signals of the defect.

**Figure 9:**
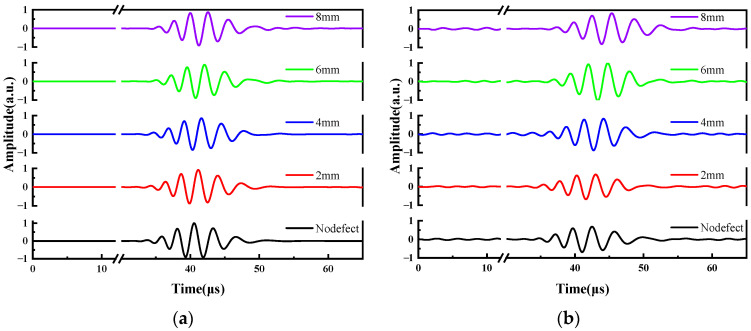
The relationship between LW transmitted signal and defect size. (**a**) FEM at a laser focus-receiving spot distance of 70 mm. (**b**) Experiment at a laser focus-receiving spot distance of 70 mm.

The correlation between defect width and LW transmission time was established through analysis of the LW transmission time across various defects. In Figure 9, various defect widths are depicted, ranging from defect free to widths of 2 mm, 4 mm, 6 mm, and 8 mm. Receiving spots were positioned at intervals of 60 mm, 70 mm, 80 mm, 90 mm, and 100 mm from the laser focus. The time difference of the LW is proportional to the increase in defect width, as displayed in Figure 9. The time differences are the same on different defects regardless of the spatial orientation of the receiving point. It implies that the propagation time of the LW remains uniform across the range of defects.

**Figure 10:**
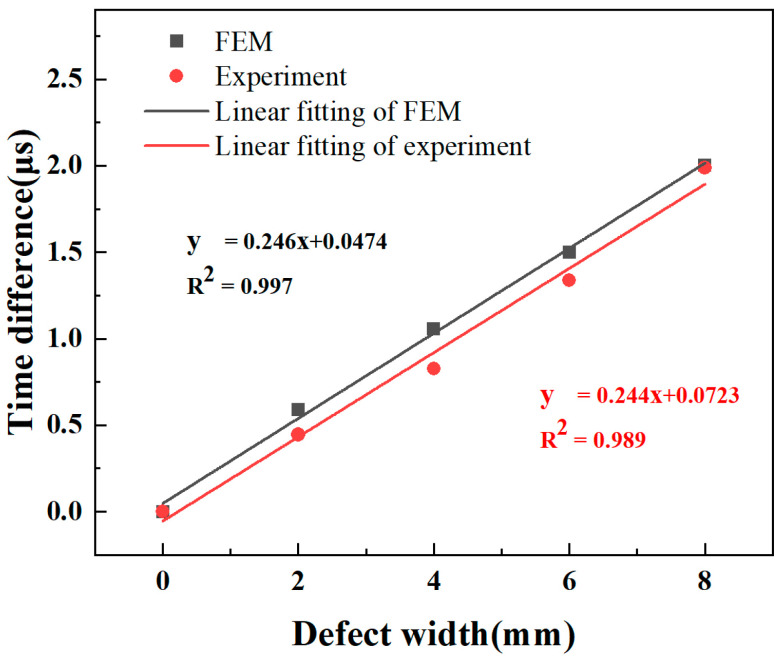
The relationships between LW transmitted signal and defect size derived by experiments and FEM.

The relationships between the LW transmitted signal and defect size are presented in Figure 9. There is a downtrend of LW displacement with defect width, causing a delay in received time. This phenomenon is attributed to both energy dissipation of LWs traversing the defects and a reduction in the velocity of the A_0_ mode. Additionally, the waveform of the LW passing through the defect undergoes changes and causes the time fluctuations of the received signal. A linear fitting analysis was conducted to describe the variations in defect width against the time difference. The linear fit, represented in Figure 10, shows the relationship between defect width and time difference. The equations of the fitted line for FEM and experimental ones are denoted, respectively:

y = 0.246 x + 0.0474,(1)

y = 0.244 x + 0.0723,(2)

Here, y is the time variation caused by the defect, and x represents the defect width. This can quantitatively characterize defects of different widths based on the equation of the fitted line. With a given detection distance, the defect width can be determined by measuring the travel time of the LW.

A defect width of 5 mm was employed to verify the method. The predicted time difference for a defect width of 5 mm is 1.276 μs according to Equation (1). In the FEM, the time difference is 1.28 μs for a 5 mm defect width. The error is 0.011 mm, corresponding to a relative error of 0.211%. The predicted time variation for a defect width of 5 mm is 1.170 μs according to Equation (2). In the experiment, the actual time difference is 1.196 μs for a 5 mm defect, resulting in a measurement error of 0.395 mm. This demonstrates the feasibility of this method for measurements of defect width.

## 5. Conclusions

A laser ultrasonic defect inspection system was developed in combination with the all-fiber heterodyne interferometer. It could detect LWs with high temporal and spatial resolution. The LW spatial-temporal wavefield of H62 brass was obtained by using the COMSOL Multiphysics software. The A_0_ mode with 370 kHz is sensitive to defect detection due to its vibrational direction and maximum energy. The size and location of defects are determined by the time difference of the transmitted wave and reflected wave, respectively. This demonstrates that the average error of defect position is 0.14% through travel time in experiments on six different receiving spots. The width of the defect is linear to the time difference of the transmitted wave, and the error can achieve 0.395 mm for a 5 mm width of defect. The observed results are consistent with those from FEM. Therefore, this validates that the feasibility of the technique can be utilized in the defect detection of thin plates.

## Figures and Tables

**Table 1:** Elastic paraments of H62 brass.

Material	Mass Density (kg/m^3^)	Young’s Modulus (GPa)	Poisson’s Ratio
H62 brass	8390	105	0.346

## Data Availability

The raw data supporting the conclusion of this article will be made available by the authors, without undue reservation.

## References

[1] Liao W., Hu Y., Liu Q. (2022). High strength and high electrical conductivity C70250 copper alloy with fibrous structure reinforced by high density, multi-scale nano-precipitates and dislocation. Mater. Sci. Eng. A.

[2] Li L., Pan D.a., Li B., Wu Y., Wang H., Gu Y., Zuo T. (2017). Patterns and challenges in the copper industry in China. Resour. Conserv. Recycl..

[3] Li Y., Wang A.J., Chen Q.S., Liu Q.Y. (2013). Influence factors analysis for the next 20 years of Chinese copper resources demand. Adv. Mater. Res..

[4] Sun X., Jie J., Wang T., Li T. (2021). Effect of two-step cryorolling and aging on mechanical and electrical properties of a Cu–Cr–Ni–Si alloy for lead frames applications. Mater. Sci. Eng. A.

[5] Zhou M., Yun X., Fu H. (2022). Producing large-scale industrially applicable high-precision copper strips by continuous extrusion with a novel U-shaped die. J. Manuf. Process..

[6] Yan X.-L., Dong S.-Y., Xu B.-S., Cao Y. (2018). Progress and challenges of ultrasonic testing for stress in remanufacturing laser cladding coating. Materials.

[7] Sanderson T. (2008). On the evaluation of residual stresses in bi-layer materials using the bent strip method. Surf. Coat. Technol..

[8] Lin J., Ma N., Lei Y., Murakawa H. (2017). Measurement of residual stress in arc welded lap joints by cosα X-ray diffraction method. J. Mater. Process. Technol..

[9] Ren S., Ren X. (2018). Studies on laws of stress-magnetization based on magnetic memory testing technique. J. Magn. Magn. Mater..

[10] Junge M., Qu J., Jacobs L.J. (2006). Relationship between Rayleigh wave polarization and state of stress. Ultrasonics.

[11] Lalik K., Dominik I., Ćwiąkała P., Kwaśniewski J. (2017). Integrated stress measurement system in tower crane mast. Measurement.

[12] Kusano M., Hatano H., Watanabe M., Takekawa S., Yamawaki H., Oguchi K., Enoki M. (2018). Mid-infrared pulsed laser ultrasonic testing for carbon fiber reinforced plastics. Ultrasonics.

[13] Davis G., Nagarajah R., Palanisamy S., Rashid R.A.R., Rajagopal P., Balasubramaniam K. (2019). Laser ultrasonic inspection of additive manufactured components. Int. J. Adv. Manuf. Technol..

[14] Zhou Z., Zhang K., Zhou J., Sun G., Wang J. (2015). Application of laser ultrasonic technique for non-contact detection of structural surface-breaking cracks. Opt. Laser Technol..

[15] Davies S., Edwards C., Taylor G., Palmer S.B. (1993). Laser-generated ultrasound: Its properties, mechanisms and multifarious applications. J. Phys. D Appl. Phys..

[16] Su Z., Ye L., Lu Y. (2006). Guided Lamb waves for identification of damage in composite structures: A review. J. Sound Vib..

[17] Giurgiutiu V., Soutis C. (2012). Enhanced composites integrity through structural health monitoring. Appl. Compos. Mater..

[18] Mitra M., Gopalakrishnan S. (2016). Guided wave based structural health monitoring: A review. Smart Mater. Struct..

[19] Cernadas D., Trillo C., Doval A.F., López O., López C., Dorrío B.V., Fernández J.L., Pérez-Amor M. (2006). Non-destructive testing of plates based on the visualisation of Lamb waves by double-pulsed TV holography. Mech. Syst. Signal Process..

[20] Bourasseau N., Moulin E., Delebarre C., Bonniau P. (2000). Radome health monitoring with Lamb waves: Experimental approach. NDTE Int..

[21] Staszewski W., Lee B., Traynor R. (2007). Fatigue crack detection in metallic structures with Lamb waves and 3D laser vibrometry. Meas. Sci. Technol..

[22] Bermes C., Kim J.-Y., Qu J., Jacobs L.J. (2008). Nonlinear Lamb waves for the detection of material nonlinearity. Mech. Syst. Signal Process..

[23] Giridhara G., Rathod V., Naik S., Mahapatra D.R., Gopalakrishnan S. (2010). Rapid localization of damage using a circular sensor array and Lamb wave based triangulation. Mech. Syst. Signal Process..

[24] Dewhurst R., Edwards C., McKie A., Palmer S. (1987). Estimation of the thickness of thin metal sheet using laser generated ultrasound. Appl. Phys. Lett..

[25] Pierce S., Culshaw B., Philp W., Lecuyer F., Farlow R. (1997). Broadband Lamb wave measurements in aluminium and carbon/glass fibre reinforced composite materials using non-contacting laser generation and detection. Ultrasonics.

[26] Dixon S., Burrows S.E., Dutton B., Fan Y. (2011). Detection of cracks in metal sheets using pulsed laser generated ultrasound and EMAT detection. Ultrasonics.

[27] Lee J.-R., Shin H.-J., Chia C.C., Dhital D., Yoon D.-J., Huh Y.-H. (2011). Long distance laser ultrasonic propagation imaging system for damage visualization. Opt. Lasers Eng..

[28] Lee J.-R., Chia C.C., Shin H.J., Park C.-Y., Yoon D.J. (2011). Laser ultrasonic propagation imaging method in the frequency domain based on wavelet transformation. Opt. Lasers Eng..

[29] Yu L., Giurgiutiu V. (2008). In situ 2-D piezoelectric wafer active sensors arrays for guided wave damage detection. Ultrasonics.

[30] Wilcox P., Lowe M., Cawley P. (2005). Omnidirectional guided wave inspection of large metallic plate structures using an EMAT array. IEEE Trans. Ultrason. Ferroelectr. Freq. Control..

[31] Liu Z., Yu H., Fan J., Hu Y., He C., Wu B. (2015). Baseline-free delamination inspection in composite plates by synthesizing non-contact air-coupled Lamb wave scan method and virtual time reversal algorithm. Smart Mater. Struct..

[32] Takeda N., Okabe Y., Kuwahara J., Kojima S., Ogisu T. (2005). Development of smart composite structures with small-diameter fiber Bragg grating sensors for damage detection: Quantitative evaluation of delamination length in CFRP laminates using Lamb wave sensing. Compos. Sci. Technol..

[33] Hosoya N., Yoshinaga A., Kanda A., Kajiwara I. (2018). Non-contact and non-destructive Lamb wave generation using laser-induced plasma shock wave. Int. J. Mech. Sci..

[34] Xue R., Wang X., Yang Q., Xu D., Sun Y., Zhang J., Krishnaswamy S. (2021). Grain size distribution characterization of aluminum with a particle swarm optimization neural network using laser ultrasonics. Appl. Acoust..

[35] Kim Y.Y., Kim E.-H. (2001). Effectiveness of the continuous wavelet transform in the analysis of some dispersive elastic waves. J. Acoust. Soc. Am..

[36] Yang L., Ume I.C. (2015). Inspection of notch depths in thin structures using transmission coefficients of laser-generated Lamb waves. Ultrasonics.

[37] Xu B., Wang M., Li P., Cheng Q., Sheng Y. (2020). Application of instantaneous parameter characteristic in active lamb wave based monitoring of plate structural health. Appl. Sci..

[38] Fedosenkov D.B., Simikova A.A., Kulakov S.M., Fedosenkov B.A. (2019). Cohen’s class time-frequency distributions for measurement signals as a means of monitoring technological processes. Steel Transl..

[39] Mahato S., Teja M.V., Chakraborty A. (2017). Combined wavelet–Hilbert transform-based modal identification of road bridge using vehicular excitation. J. Civ. Struct. Health Monit..

[40] Wu Z., Huang N.E. (2009). Ensemble empirical mode decomposition: A noise-assisted data analysis method. Adv. Adapt. Data Anal..

[41] Yan G. (2013). A Bayesian approach for damage localization in plate-like structures using Lamb waves. Smart Mater. Struct..

[42] Soltani P., Akbareian N. (2014). Finite element simulation of laser generated ultrasound waves in aluminum plates. Lat. Am. J. Solids Struct..

[43] Zhao Y., Shen Z., Lu J., Ni X., Cui Y. (2009). Laser-induced circumferential waves on hollow cylinder and their interaction with defects by finite element method. Indian J. Phys..

[44] Zeng W., Qi S., Liu L., Yao Y. (2019). Research on laser-generated Rayleigh waves with angled surface crack by finite element method. Optik.

[45] Jian X., Dixon S., Quirk K., Grattan K. (2008). Electromagnetic acoustic transducers for in-and out-of plane ultrasonic wave detection. Sens. Actuators A Phys..

[46] Yang Y., Peng Z., Zhang W., Meng G. (2019). Parameterised time-frequency analysis methods and their engineering applications: A review of recent advances. Mech. Syst. Signal Process..

[47] Lee J.-Y., Chen H.-Y., Hsu C.-C., Wu C.-C. (2007). Optical heterodyne grating interferometry for displacement measurement with subnanometric resolution. Sens. Actuators A Phys..

[48] Wu C.-C., Hsu C.-C., Lee J.-Y., Chen H.-Y., Dai C.-L. (2008). Optical heterodyne laser encoder with sub-nanometer resolution. Meas. Sci. Technol..

[49] Das S., Schill A., Liu C.-H., Aglyamov S., Larin K.V. (2020). Laser-induced elastic wave classification: Thermoelastic versus ablative regimes for all-optical elastography applications. J. Biomed. Opt..

[50] Wang Z., Zhang L., Wang S., Xue N., Peng F., Fan M., Sun W., Qian X., Rao J., Rao Y. (2016). Coherent Φ-OTDR based on I/Q demodulation and homodyne detection. Opt. Express.

[51] Tu X., Sun Q., Chen W., Chen M., Meng Z. (2014). Vector Brillouin optical time-domain analysis with heterodyne detection and IQ demodulation algorithm. IEEE Photonics J..

[52] Chen H., Xu K., Liu Z., Ta D. (2022). Sign coherence factor-based search algorithm for defect localization with laser generated Lamb waves. Mech. Syst. Signal Process..

